# Closing the loop: autonomous intelligent control for hypoxia pre-acclimatization and high-altitude health management

**DOI:** 10.1093/nsr/nwaf071

**Published:** 2025-03-08

**Authors:** Dawei Shi, Jing Chen, Meitong Li, Lingling Zhu, Xunming Ji

**Affiliations:** School of Automation, Beijing Institute of Technology, Beijing 100081, China; School of Automation, Beijing Institute of Technology, Beijing 100081, China; School of Automation, Beijing Institute of Technology, Beijing 100081, China; Institute of Military Cognition and Brain Sciences, Academy of Military Medical Sciences, Beijing 100850, China; Department of Neurosurgery, Xuanwu Hospital, Capital Medical University, Beijing 100053, China

**Keywords:** pre-acclimatization, high-altitude health management, wearable sensors, physiological closed-loop control systems

## Abstract

Hypobaric hypoxia at high altitudes threatens the health of high-altitude residents. The development of effective methods to guarantee the safety of frequent human activities in high-altitude locations is therefore needed. Pre-acclimatization at sea level is an effective approach to mitigate subsequent altitude sickness for rapid ascent, which offers a viable substitute to on-site acclimatization, minimizes the associated risks that are linked to prolonged exposure in high-altitude environments and can be personalized to individual hypoxic responses. Another critical aspect to prevent long-term physical damage is personalized health management at high altitudes, which is enabled by the emerging technologies of wearable sensors, the Internet of Medical Things and artificial intelligence. In this review, we outline the progress in pre-acclimatization and high-altitude health management, as well as the understanding of physiological mechanisms under hypoxia, highlighting the important role that is played by wearable sensors and physiological closed-loop control systems in developing intelligent personalized solutions. We also discuss the challenges and prospects of deploying autonomous intelligent monitoring and control in high-altitude health management.

## Teaser

This review introduces advancements in pre-acclimatization and health management at high altitudes, emphasizes the important role of wearable sensors and autonomous intelligent control in high-altitude healthcare, and proposes a promising personalized closed-loop control framework for oxygen saturation regulation.

## INTRODUCTION

High altitude (>2500 m above sea level), which is normally associated with decreased oxygen availability, lower atmospheric pressure and lower temperatures, can significantly cause human metabolism to deviate from the normal equilibrium [1]. The physiological responses can transform into pathological development under severe hypoxia, which can lead to acute mountain sickness (AMS), high-altitude pulmonary edema (HAPE) and high-altitude cerebral edema (HACE) for lowlanders who are ascending to high altitudes [2]. Related effects in the human body include cognitive function impairment, physical performance limitation, as well as irreversible damage due to subsequent chronic alterations [3]. Current estimates suggest that >140 million individuals worldwide reside or work at elevations of >2500 m [4] and 1 million people are attracted to high altitudes annually, which gives rise to a substantial public health issue at high altitudes.

Alleviation of the consequences of these health issues is the ultimate goal of high-altitude medicine in which pre-acclimatization at sea level and health management at high altitudes are two critical measures. Pre-acclimatization aims to accelerate the adaptation process of lowlanders who are ascending to high altitudes and stimulates a series of stress responses in the body by exposing a subject to controlled low-oxygen conditions [5]. Health management at high altitudes, on the other hand, mainly focuses on the preservation of normal physiological homeostasis for both high-altitude residents and travelers who stay at such locations for long periods, for which additional oxygen supply is the key intervention approach to prevent progressive deterioration [6]. Particularly, with the advancement in oxygen enrichment technology, individuals who are ascending to high altitudes are increasingly exposed to alternating hypoxic and hyperoxic environments, which complicates high-altitude health management and also underscores the importance of oxygen-saturation regulation of the human body. The effectiveness of pre-acclimatization has been widely evaluated among different populations under various experimental settings, which were mainly designed based on subjective clinician experience. It is still not clear how to systematically design the pre-acclimatization strategies with guaranteed safety and efficiency [7]. Besides, even with the aid of pre-acclimatization before ascent, persistent health management is still critically challenging due to the huge intersubject variability in the hypoxic physiological responses and the diversity of daily activities at high altitudes.

**Figure 1.**
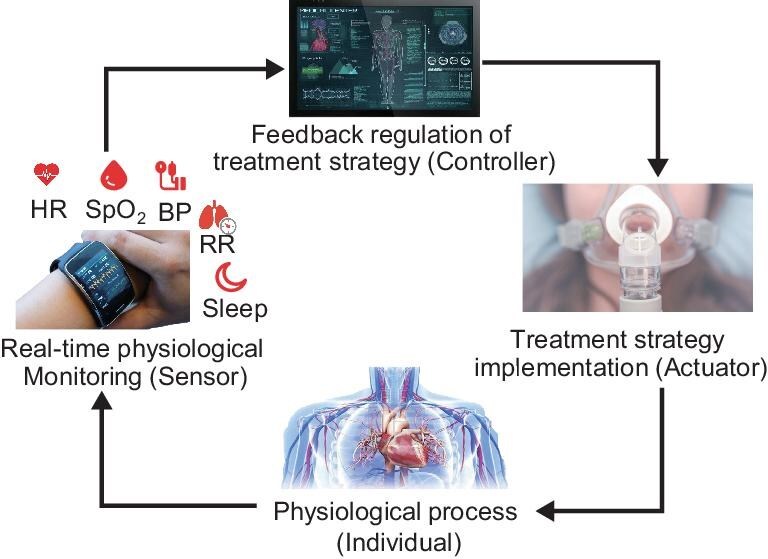
Schematic diagram of physiological closed-loop control systems.

**Figure 2.**
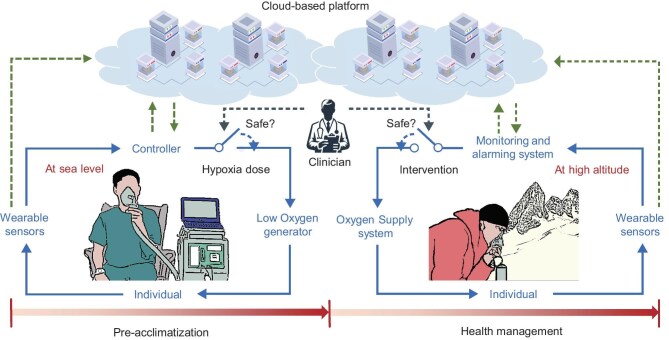
Personalization framework for pre-acclimatization and high-altitude health management. During pre-acclimatization, multiple physiological parameters are monitored through wearable devices, which are wirelessly transmitted to the controller for decision-making. Then, the hypoxia dose is determined through the designed closed-loop algorithm and it is performed under the supervision of the clinician. After going to the high altitude, the continuously monitored signals are collected and analysed for an early alarm of potential risks. Proper suggestions can be immediately given through mobile devices and the remote clinician diagnosis and intervention are available in emergency rooms. In this process, all the personalized medical data are synchronously stored in the cloud for retrospective analysis.

A promising approach to addressing these challenges is to consider physiological closed-loop control [8] (see Fig. 1), which is an emerging application of systems and control theory in healthcare. A physiological closed-loop control system integrates sensors, controllers and actuators with the human physiological processes to achieve intelligent decision-making based on feedback mechanisms. To enable such systems, real-time physiological monitoring that is empowered by wearable sensors plays a critical role. By continuously tracking multiple physiological parameters that are associated with high-altitude illnesses, the control algorithm can dynamically regulate the hypoxia or supplied oxygen doses based on the individual's current condition to enable accurate and personalized decision-making (see Fig. 2).

**Figure 3.**
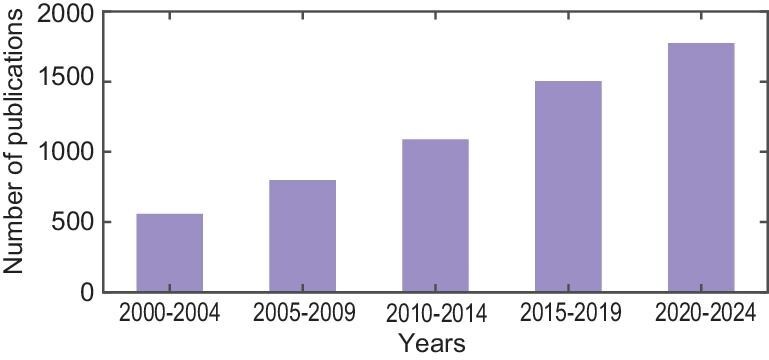
Number of publications in oxygen-saturation regulation for high-altitude health management (2000–24).

**Figure 4.**
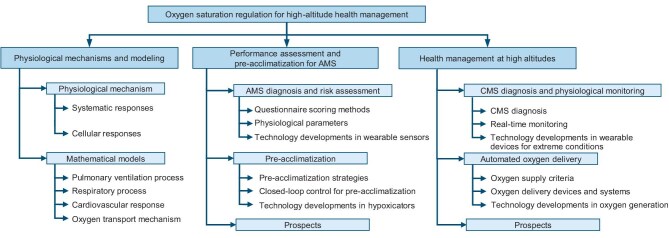
Structure of the paper on oxygen-saturation regulation for high-altitude health management.

Motivated by the increasing attention on high-altitude medicine (see Fig. 3) and the lack of a systematic review of the recent developments, the objective of this paper is to characterize essential aspects of oxygen-saturation regulation for high-altitude health management and, more importantly, to offer a closed-loop control scheme for personalizing high-altitude medical practices. This paper covers hypoxia-induced physiological mechanisms and modeling, performance assessment and pre-acclimatization approaches for AMS, and long-term health management systems at high altitudes. The prospect of personalized healthcare with the support of non-invasive wearable sensors and intelligent control algorithms is also emphasized. The target audience of this work consists of both researchers and healthcare providers who are working on oxygen-saturation regulation for high-altitude health management and researchers who are working on the more generic topic of closed-loop drug delivery. The structure of this paper is summarized in Fig. 4.

For the purpose of the study, an exhaustive literature search was carried out in various online databases, including Google Scholar, IEEE Xplore, ScienceDirect and PubMed. The search mainly focused on peer-reviewed journal articles and conference proceedings that were published between 2000 and 2024, which was performed by concatenating terms such as ‘pre-acclimatization’, ‘intermittent hypoxic exposure’, ‘oxygen supply’, ‘physiological monitoring’, ‘acute mountain sickness’, ‘remote ischemic preconditioning’ and ‘hypoxia’, and terms related to high altitudes. The selected papers prioritized results that highlighted significant technical advancements or clinical evaluations. Information from the selected literature was extracted according to predefined categories (Fig. 4), which were developed based on our prior research effort and experience toward closed-loop personalized pre-acclimatization and high-altitude health management [9–14] and the identified references therein. These categories were also refined through collaborative discussions between the authors, integrating interdisciplinary expertise from both high-altitude medicine and control science.

## HYPOXIA-INDUCED PHYSIOLOGICAL MECHANISMS AND MODELING

**Figure 5.**
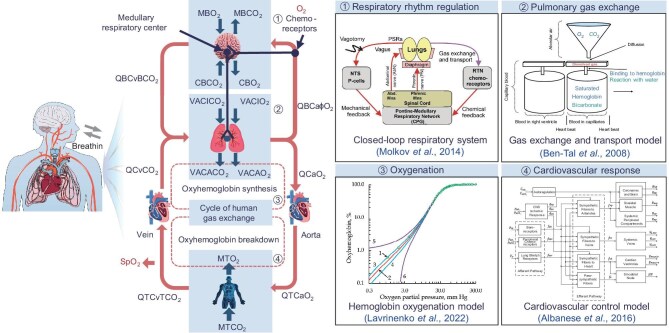
The respiratory process and physiological modeling under hypoxia. MBCO_2_: brain tissue CO_2_ production (liters/minute), MBO_2_: brain tissue O_2_ production (liters/minute), CBCO_2_: brain tissue CO_2_ concentration (volumetric fraction), CBO_2_: brain tissue O_2_ concentration (volumetric fraction), VA: average alveolar volume input (liters), CICO_2_: inspired CO_2_ concentration (saturated with H_2_O vapor at body temperature), CIO_2_: inspired O_2_ concentration (saturated with H_2_O vapor at body temperature), CACO_2_: alveolar CO_2_ concentration (volumetric fraction), CAO_2_: alveolar O_2_ concentration (volumetric fraction), QB: cerebral blood flow (liters/minute), CvBCO_2_: brain–venous CO_2_ concentration (volumetric fraction), Ca$\phi $O_2_: brain–arterial O_2_ concentration (volumetric fraction), Q: cardiac output (liters/minute), CaO_2_: alveolar–arterial O_2_ concentration (volumetric fraction), CvCO_2_: alveolar–venous CO_2_ concentration (volumetric fraction), QT: body blood flow (liters/minute), CvTCO_2_: body–venous CO_2_ concentration (volumetric fraction), MTCO_2_: body tissue CO_2_ production (liters/minute), MTO_2_: body tissue O_2_ production (liters/minute). This figure is partly from [15].

In this section, we briefly introduce the key physiological responses that are induced by hypoxia, as well as existing models of the corresponding processes (see Fig. 5).

Hypobaric and hypoxic conditions directly impede the normal pulmonary gas exchange, which involves the intake of oxygen and the expulsion of carbon dioxide. The blood oxygen saturation in the body decreases significantly and the hyperventilation attempts to relieve the disorders that are caused by the low-oxygen environment. The main causes of hyperventilation are the limited diffusion capability of oxygen from the alveoli to the arteries that is caused by reduced pressure differentials and the unequal distribution of ventilation and perfusion. Generally, an increasing elevation of altitude is followed by an acute hypoxic ventilatory response (HVR) within a few minutes and a hypoxic ventilatory drop within several tens of minutes. The chronic hypoxic ventilatory acclimatization can be observed after months, which is partly attributed to the respiratory regulation of the central nervous system (CNS).

The CNS generates the basic respiratory rhythms via the central respiratory pattern generator (CPG) within the brainstem. The breathing amplitudes, frequency and patterns are controlled via chemical and mechanical feedback [16]. When exposed to acute hypoxia, the chemoreceptors detect a drop in oxygen saturation and then the CNS responds by increasing the regulatory frequency and tidal volume, and potentially altering the respiratory pattern, which ultimately achieves the homeostatic regulation of inspiration and exhalation. However, its function may be partly impaired due to abrupt hypoxia, causing motor execution and cognitive function impairments [17].

Related mathematical models of the pulmonary ventilation process have been successively proposed, which usually take the respiratory rate, lung volume, external oxygen and carbon dioxide concentrations as inputs and the oxygen and carbon dioxide concentrations in arteries and veins as outputs. Regarding the lung as a flexible container, Ben-Tal *et al*. constructed non-linear single-compartment models of lung gas exchange, which can describe the main kinetics of oxygen and carbon dioxide, but ignore the heterogeneous lung tissue structure [18]. By using the multifield and multiscale coupled modeling method, Roth *et al*. designed mathematical models that focused more on the local perfusion and regional differences; they were developed based on the airway and blood vessel data of real patients and the effectiveness was validated through numerical simulations [19]. Furthermore, considering the effects of blood pressure (BP) changes and blood flow velocity in capillaries of alveolar tissue, Zurita and Hurtado established a 3D capillary model to achieve more accurate simulation of the gas exchange and perfusion process [20]. Unlike these complex models that describe the human respiratory dynamics in detail, a simple but useful control-oriented model was built based on the experimental data from large mammals to help peritoneal oxygenation technology development [21]; it only considers the oxygen and ignores the carbon dioxide changes.

Considering the effect of the CNS, Molkov *et al.* constructed a computational model of the closed-loop respiratory system that was oriented for neuronal conductance processes, which can describe the brainstem respiratory network that controls pulmonary ventilation. This model focuses on hypercapnia and active expiration in the hypoxia condition [16]. In [22], optogenetic experiments that selectively stimulate the post-inspiratory inhibitory neurons (PiNs) were performed to explore the role of the PiNs in the CPG. The observed phenomenon that active inhibition of PiNs prolongs the post-inspiratory phase was mathematically described and was integrated into Molkov's model to provide a more accurate description of respiratory regulation under hypoxic conditions, particularly in the presence of hypercapnia and active expiration. Concentrating on respiratory pattern generation and switching, Diekman *et al*. described the CPG by using the Butera–Rinzel–Smith model. The bistability and robustness of the model were discussed based on system dynamics analysis [23].

Apart from obtaining more oxygen through hyperventilation, the body also increases the oxygen affinity of hemoglobin, the transportation of which involves the cardiovascular system. The cardiovascular system mainly consists of the heart and the blood vessels, whose functions are to power blood circulation and to transport nutrients, wastes and other substances, respectively. To compensate for the lack of oxygen, the heart rate (HR) increases during the first few days of the acute ascent to high altitude, as well as the myocardial contractility and cardiac output [24]. Subsequently, the number of erythrocytes gradually increases to further enhance the oxygen transport capacity [25]. Besides, the direct vasodilator effect that is caused by hypoxia maintains or even reduces the BP [7] but the BP will increase with days or weeks of hypoxia exposure because of the increased HR, the vasoconstriction and the sympathetic modulation [26].

To describe the acute cardiovascular response to isocapnic hypoxia, Ursino and Magosso proposed a mathematical model that covers the processes of heart pulsation, systemic circulation, pulmonary circulation and oxygen response [27]. On this basis, an integrated model that includes respiratory, cardiovascular and sleep–wake regulation was constructed by Cheng *et al.* and can assist in medical interventions [28]. In order to reflect the interaction between the cardiovascular system and the respiratory system, Albanese *et al*. [29] improved the thoracic cavity structure in the Ursino and Magosso model and adjusted the parameters by combining the results in Cheng *et al.* [28].

The oxygen dissociation curve describes the relationship between the hemoglobin oxygen saturation and the partial pressure of oxygen (PO_2_). The curve shifts left when the oxygen affinity capacity increases, which is affected mainly by temperature, pH, partial pressure of carbon dioxide and 2,3-diphosphoglycerate (2,3-DPG). In high-altitude environments, the oxygen affinity is reduced due to the 2,3-DPG that is produced by the anaerobic glycolysis of the increased erythrocytes. The rises in blood pH and oxygen affinity that are caused by hyperventilation can relieve this symptom, but this probably results in a slight respiratory alkalosis [30]. The Hill equation is a mathematical model that is commonly used to describe oxygen dissociation curves and contains critical parameters with unclear physical meaning. To solve this problem, Lavrinenko *et al.* improved the Hill equation by transforming the power of PO_2_ from a constant value to a variable that is dependent on the PO_2_ based on Gauss and Lorentz distributions, which is more physiologically interpretable [31].

To maintain oxygen homeostasis, a range of responses to hypoxia are activated by stable hypoxia-inducible factors (HIFs) at the cellular level [7]. HIFs are transcription factors that consist of either HIF-1$\alpha $ or HIF-21$\alpha $ and HIF-1$\beta $/ARNT subunits, which promote the expressions of target genes to enhance oxygen delivery and utilization. Related pathways under the transcriptional control of HIFs mainly include erythropoiesis, angiogenesis and metabolic reprogramming [32]. Specifically, HIF-1$\alpha $ and HIF-2$\alpha $ upregulate the erythropoietin (EPO) and the vascular endothelial growth factor, which are involved in red blood cell production and blood vessel formation, respectively, to improve tissue oxygenation [33]. For metabolic reprogramming, HIF-1$\alpha $ induces the expression of glycolytic enzymes to accelerate the production of adenosine triphosphate under low-oxygen conditions. Simultaneously, the mitochondrial oxygen consumption is suppressed to reduce the mitochondrial production of reactive oxygen species that influences stabilization of the HIFs [34]. Recently, the role of HIF-2$\alpha $ in inducing oxygen chemosensitivity has also been explored, which reflects the essential function of HIF-2$\alpha $ in augmenting HVR after acclimatization to hypoxia [35]. For highlanders, strong evidence of population-specific selection was identified in the HIF genes. Polymorphisms that are related to HIF-pathway elements are specific for certain high-altitude populations (e.g. Tibetan highlanders), while others (e.g. Andean highlanders) are less protected from chronic mountain sickness (CMS) [36].

## PERFORMANCE ASSESSMENT AND PRE-ACCLIMATIZATION FOR AMS

Early prevention and diagnosis of AMS are critical for avoiding or alleviating deterioration to more severe consequences such as HAPE and HACE. In this section, we review the major existing approaches for AMS risk assessment and pre-acclimatization.

### Diagnosis and risk assessment of AMS

#### Questionnaire scoring methods

A persuasive indicator for the risk assessment of AMS is a prerequisite for developing optimal pre-acclimatization strategies. One of the most-recognized standards is the Lake Louise score (LLS), which is widely used in evaluating AMS severity through a questionnaire and a scorecard. Subjects are asked to self-report four related symptoms (headache, gastrointestinal symptoms, fatigue/weakness and dizziness/light-headedness). Each symptom is scored on a scale from 0 to 3 and the severity of the AMS is diagnosed based on the total score (mild AMS: 3–5, moderate AMS: 6–9, severe AMS: 10–12) [64]. Based on the 67-item environmental symptom questionnaire (ESQ-III), 11 items are extracted to form a simplified ESQ-C scoring system. Each symptom is scored on a scale of 0–5; a weighted score of cerebral symptoms of >0.7 is classified as AMS [65]. Besides, the Chinese altitude sickness scoring system, which was unanimously established by the Third Ad Hoc Committee on High Altitude Illnesses of Chinese Medical Association, considers more non-specific symptoms compared with the LLS, results in a higher prevalence of AMS. On the one hand, highly non-specific symptoms cause an ongoing debate about AMS diagnosis. Specifically, considering the weak correlation between sleep disturbance and AMS, the ‘sleep disturbance’ symptom was removed from the earliest version of the LLS [66]. In [67], it was also suggested that headache should not be a required symptom for AMS diagnosis. On the other hand, questionnaire scoring methods are essentially subjective and may lead to false-positive or false-negative AMS diagnosis, which highlights the importance of evidence-based continuous monitoring of physiological parameters and vital signs in AMS detection and diagnosis.

#### Physiological parameters

Several hypoxic-related physiological parameters are promising indicators for AMS. These variables can objectively track the dynamics of AMS development in an easily accessible and non-invasive way.

##### Oxygen saturation.

Oxygen saturation quantifies the proportion of oxygen-bound hemoglobin to the total hemoglobin in the blood. The peripheral oxygen saturation (SpO_2_) is usually measured through a pulse oximeter or a wristband and its effectiveness in predicting AMS susceptibility has been evaluated in various experiments (see Table 1). Overall, the association between SpO_2_ and AMS was reported in many of the experiments, although some studies did not observe such a correlation. Key factors that may influence the results should not be overlooked. First, the data quality can be degenerated by different interference (e.g. bright ambient light, excessive movement, poor probe positioning, vasoconstriction induced by low temperature [68]), resulting in large measurement errors if the measurement process is not strictly conducted under professional supervision. This constitutes one of the reasons for the lack of comparability between different studies. Apart from external disturbances, discrepancies in skin pigmentation and respiratory intensity can also strongly affect the measurement accuracy and are unavoidable in the experiments. Second, the AMS susceptibility prediction analysis is usually performed only based on one-shot SpO_2_ or mean SpO_2_, which is the method that is most commonly used but has its limitations. The dynamic properties cannot be properly captured by a static value. Actually, the SpO_2_ level fluctuates with diurnal circadian rhythms. In [69], the SpO_2_ level featured an increase after 2-h intervals of uninterrupted sleep and the SpO_2_ at night was significantly lower than the SpO_2_ at rest in the morning. This time-varying nature renders static or intermittent measurements unstable and unreliable, which means a robust method to evaluate the connection between oxygen saturation and AMS is needed. Finally, the experimental set-up is a critical factor that affects the results. For specific altitudes, different SpO_2_ thresholds of AMS diagnosis were given under various predesigned scenarios (e.g. resting, submaximal exercise or sleeping). It is worth noting that, although the inconsistent standards of experiment design that are based on expert experiences prevent rational comparative analysis to some extent, the persuasiveness of using the SpO_2_ in AMS susceptibility prediction is strengthened by these evaluation results, which were obtained from different population distributions and altitude settings [44,59].

**Table 1.** Summary of AMS detection by using physiological parameters.

No.	Ref.	Indicator	Maximum altitude	Case	LLS threshold	Main analysis method	Conclusion
1	[37]	SpO_2_, HR, BP and CI for 5 min after 3 h of rest	3775 m	4/7	$\ge $ 3	Difference analysis	SpO_2_: no; HR: yes; BP: no; CI: yes
2	[38]	SpO2, VE	6022 m	23/16	$\ge $ 5	Pearson correlation	SpO_2_: yes; VE: yes
3	[39]	Fasting SpO_2_, HR and RR after XEST	5300 m	244/88	$\ge $ 3 and $\ge $5	Difference analysis, logistic regression	SpO_2_: yes; RR: yes
4	[40]	Resting SpO_2_ and HR, mean VE after stabilization	3480 m	13/27	$\ge $ 3	Pearson correlation, multivariate stepwise logistic regression	SpO_2_: yes; HR: yes; VE: yes
5	[41]	Mean SpO_2_, HR, rScO_2_ and cerebral oxygenation five times	3883 m	6/5	$\ge $ 3	Difference analysis, Spearman correlation	SpO_2_: no; HR: yes; rScO_2_: yes; CI: yes
6	[42]	SpO_2_ and HR after at least 12 h of fasting	5085 m	20/8	$\ge $ 3	ROC curve	SpO_2_: yes; HRv: yes
7	[43]	ECG for 15 min before sleep and after awakening	3150 m	19/20	$\ge $ 3	Spearman correlation, time and frequency-domain analysis	HRv: yes
8	[44]	SpO_2_ and HR for 2 min outdoors or in rooms with opened windows	5500 m	100/104	$\ge $ 3 and $\ge $6	Spearman correlation	SpO_2_: debatable
9	[45]	SpO_2_ and HR for 15 min in a seated position	4400 m	461/291 104/163	$\ge $ 3 and $\ge $6	Difference analysis, stepwise logistic regression	SpO_2_: yes; HR: yes
10	[46]	SpO_2_, HR, BP, ECG and Doppler after 30 min of rest	3700 m	84/66	$/$	Difference analysis, PCA, regression analysis	SpO_2_: no; HR: yes
11	[47]	Mean SpO_2_ and cardiorespiratory parameters after 30 min of exposure	4500 m	34/21	$> $ 3	Difference analysis, sequential logistic regression	SpO_2_: yes; TV and fB: yes
12	[48]	SpO_2_ and BP after 15 min of rest	3700 m	128/204	$\ge $ 3	Difference analysis	SpO_2_: yes; BP: yes
13	[49]	SpO_2_ and HR for 24 h	4559 m	24/36	$\ge $ 3 and $\ge $5	Difference analysis, Pearson correlation, ROC curve	SpO_2_: yes; HR: no
14	[50]	Stable SpO_2_ and HR for 1 min	5640 m	33/23	$\ge $ 3	Difference analysis	HRv: yes
15	[51]	SpO_2_ and HR after 15 min of rest	3952 m	258/529	$\ge $ 4	Difference analysis, multivariate logistic regression, ROC curve	SpO_2_: no; HR: no
16	[52]	SpO_2_, HR and BP after 5 min of rest and 2 min of exercise	5200 m	32/6	$\ge 3$	Difference analysis, Spearman correlation	SpO_2_: yes; HR: yes
17	[53]	SpO_2_ and HR after 15 min of rest, ECG for 5 min	6300 m	24/12	$\ge $ 3	Difference analysis, Pearson correlation	HRv: yes
18	[54]	BP after 2 min of rest, PPG three times in 1 min	4770 m	7/10	$\ge $ 3	Difference analysis, Pearson correlation	BP: no
19	[55]	SpO_2_, hematocrit	5400 m	16/31	$\ge $ 4	Stepwise regression, linear mixed-effect model, ROC curve	SpO_2_: yes; hematocrit: yes
20	[56]	Sleep SpO_2_ for 5 min	3800 m	4/3	$\ge $ 4	Difference analysis	SpO_2_: yes
21	[57]	SpO_2_, HRv and BP for 10 min after 5 min of rest	4380 m	21/20	$> $ 2	Correlation analysis, ROC curve, frequency-domain analysis	SpO_2_: yes; BP: yes; HRv: no
22	[58]	SpO_2_, resting ECG for 5 min after trekking	3440 m	7/25	$> $ 4	Frequency-domain analysis	SpO_2_: no; HRv: yes
23	[59]	Mean SpO_2_ four times in 15 s after walking	5300 m	39/44	$\ge $ 3	Difference analysis, sensitivity, specificity	SpO_2_: yes
24	[60]	SpO_2_, HR, fB and CBF for 30 min after 10 min of rest	4200 m	6/6	$> $ 4	Difference analysis	SpO_2_: no; CBF: no
25	[61]	SpO_2_, HR and ECG for 10 min after 2 h of rest	3456 m	12/9	$> $ 2	Maximum entropy method, difference analysis, regression analysis	SpO_2_: no; HRv: yes
26	[62]	SpO_2_ for 1 min after 20–30 min of rest	3659 m	63/87	$> $ 2	Difference analysis, stepwise logistic regression analysis	SpO_2_: yes
27	[63]	SpO_2_	6194 m	43/59	$> $ 2	Correlation analysis	SpO_2_: yes

SpO_2_ = peripheral oxygen saturation, HR = heart rate, BP = blood pressure, HRv = heart rate variability, CI = cardiac index, VE = minute ventilation, RR = respiratory rate, rScO_2_ = cerebral oxygen saturation, ECG = electrocardiogram, TV = tidal volume, fB = frequency of breathing, PPG = photoplethysmographic, IP = intracranial pressure, CBF = cerebral blood flow, ROC curve = receiver operating characteristic curve, PCA = principle component analysis. Case represents AMS/N-AMS subjects. The predictive and non-predictive conclusions of indicators are denoted by ‘yes’ and ‘no’, respectively.

##### HR-related parameters.

In the 27 clinical studies that are reported in Table 1, 15 studies that involved 2373 subjects reported an association between SpO_2_ and AMS, while the correlation of AMS with HR was only reported in 6 studies with 1269 subjects. These results indicate that the correlation between HR and AMS is less significant than that between SpO_2_ and AMS, which was also reported in [70]. Despite this observation, an obvious increase in HR can be observed after reaching high altitudes [24]. HR is measured and utilized in a similar way to SpO_2_, but it has higher accuracy for its simpler measurement principles and higher technical maturity. From this perspective, HR-based AMS diagnosis and prediction may have more advantages in practical applications. Some investigations reported the effectiveness of ${\mathrm{\Delta }}$HR (differences before and after high-altitude exposure) and heart rate variability (HRv). In [45], a total of 1019 subjects were assigned to either the acute exposure group (from 500 to 3700 m within 2.5 h) or the pre-acclimatization group (ascended to 4400 m from 3650 m within 3 h after the pre-acclimatization for 33 days). In this setting, ‘${\mathrm{\Delta }}$HR > 25, SpO_2_ < 88%’ and ‘${\mathrm{\Delta }}$HR > 15, SpO_2_ < 86%’ were found to be the independent factors of AMS development for the acute exposure group and the pre-acclimatization group, respectively. Several results concluded the possibility of HRv for AMS susceptibility prediction, but the exact association is still unclear. From the data analysis aspect, both ${\mathrm{\Delta }}$HR and HRv are important features that are calculated based on continuous electrocardiography (ECG) signals, which indicates the possibility of using end-to-end intelligent algorithms to reveal unexplored information for AMS risk assessment.

##### Other parameters.

Although hypoxia can cause high BP, the capability of BP for AMS prediction has not yet been clearly demonstrated in existing studies. The cerebral blood flow (CBF), respiratory rate and sleep-related parameters have also been considered in some investigations, but no extensive evidence supports their effectiveness. These parameters may not be the independent predictive factors of AMS, but can offer available auxiliary information to mitigate the non-specificity of SpO_2_ and HR variations.

##### Multiple-parameter-based AMS assessment.

Although multiple parameters are measured during experiments, existing studies mainly explore their separate predictive potentials based on stable or mean values. Statistical analysis methods are often utilized to test the indicator differences between AMS+ and AMS– groups or to determine whether there is an association between the indicators and LLS through correlation analysis or regression models. However, to enhance the accuracy and robustness of AMS assessment, the capturing of various and critical features from multiple continuous physiological parameters through data fusion is rather important, as it can provide a more comprehensive understanding of the body's response to hypoxia from different aspects.

By leveraging different types of data, machine-learning (ML) technologies are capable of identifying complex, non-linear relationships and dynamic patterns among variables that may remain uncovered by traditional statistical methods. In [71], physiological and environmental data were integrated to build AMS prediction models that were based on four different ML approaches. All models achieve high classification performance with areas under the curve of >0.99. A slow feature-based long short-term memory network that integrates heterogeneous data was proposed in [72] to predict AMS susceptibility and reaches a classification accuracy of 85.71%. Furthermore, to achieve extensive validation, different ML approaches were employed in [73] based on a large dataset that was collected from 10 438 subjects; random forest was shown to yield compelling results with a classification accuracy of 78.12%, which underscores the substantial promise of ML in advancing AMS prediction.

#### Technology development of wearable sensors

##### Oxygen-saturation sensors.

Oxygen-saturation sensors measure the SpO_2_ level mainly through photoplethysmography (PPG) technology, which is operated according to the light-absorption differences between oxygenated and deoxygenated hemoglobin. Such sensors include two LED lights with different wavelengths that are generally emitted to a fingertip and a photodetector to measure the corresponding transmitted light intensity. The SpO_2_ can be obtained by analysing the light-intensity fluctuations that are caused by changes in blood volume with each heartbeat.

**Figure 6.**
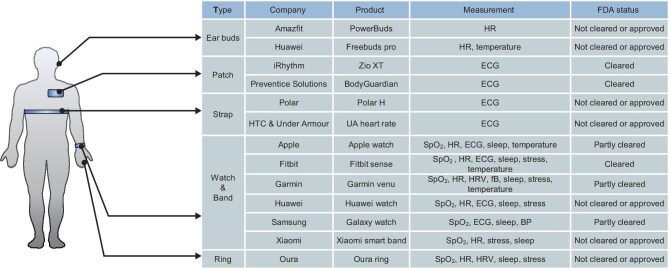
Mainstream wearable devices in different product forms on the market. Pictures of the above products are from the Internet.

A number of signal-processing methods have been proposed to mitigate the motion artifact and calibrate measurement errors that are caused by skin color, lighting, etc. [74]. Although fingertip-based oximeters can obtain higher signal amplitudes, this configuration restricts hand movements and is not optimal for long-term usage. For this reason, other measurement positions have been considered and oxygen-saturation sensors are designed with various portable product forms (smart watches/bands, smart rings, ear-clip/forehead pulse oximeters). Some typical products are shown in Fig. 6.

Several studies have evaluated the performance of wearable oxygen-saturation sensors from different perspectives. A comparative study of the Apple Watch Series 6 and two conventional commercial oximeters devices concluded the effectiveness of the Apple smart watch, based on data from patients with lung diseases [75]. Another investigation evaluated the accuracy of SpO_2_ measurements from different consumer smartwatches (Apple Watch Series 7, Garmin Venu 2S, Garmin Fenix 6 Pro and Withings ScanWatch), in which the performance that was closest to the reference standard was found in the Apple Watch Series 7 [76]. Other commercial products include the Oura Ring, NellcorTM SpO_2_ Forehead sensor and 8000Q2 Reusable Ear Clip Pulse Oximetry sensor, etc. Besides, considering the area-scaling complexity that is caused by wearables with rigid form, many flexible and comfortable sensors have been designed to increase skin contact areas for enhanced sensor performance by using all-organic materials [77]. However, the reduced accuracy of SpO_2_ measurements when using wrist-worn oximeters at high altitudes was also reported [78], which raises doubts about using SpO_2_ alone for AMS risk assessment.

##### HR-related sensors.

Commercial products for HR monitoring are mainly designed based on ECG or PPG technology. The PPG-based HR value can also be provided by the pulse oximeters mentioned above, but with different signal-processing methods from that of SpO_2_. A recent study evaluated the performance of four mainstream wrist-worn devices during maximal stress testing [79], three of which are cleared by the food and drug administration (FDA) (Apple Watch, Samsung G2 and Fitbit Charge). The Tomtom Runner Cardio and Apple Watch exhibited reliable measurements whereas Samsung G2 and Fitbit Charge tended to underestimate the HR levels. Besides, the relatively higher measurement stability of HR was found at high altitudes compared with SpO_2_ [78].

Differently from PPG, ECG records the tiny electrical activities of the heart through electrodes to measure not only HR and HRv, but also heart-rhythm indicators on the basis of heartbeat change patterns, which is regarded as a clinical-grade gold standard. Chest-strap monitors and ECG patches are typical ECG-based products. The former has higher accuracy than wrist-worn devices [80], but with long-term inconvenience compared with ECG patches. FDA-cleared patches include Zio Patch, BioTel Heart and Nuvant MCT, to mention a few.

##### Other sensors.

BP can be estimated based on the pulse transit time or pulse wave velocity through PPG and ECG [81] or directly measured by using strain and pressure sensors [82]. Several systems that embed self-calibrated modules or ML techniques have been designed to improve measurement performance. For cerebral ischemia and hypoxia monitoring, CBF and cerebral blood oxygen saturation are the key indicators that are measured through the near-infrared spectroscopy technique [83], which enables the detection of cerebral vascular responses that are caused by AMS. Sleep quality at high altitudes was monitored by using a polysomnography-based device [84], which integrated electroencephalography (EEG), electromyography (EMG) and electrooculography (EOG). An actigraph was later used in [85], which is correlated with polysomnography in sleep estimation. Besides, emerging gas sensors [86], breath sensors [87] and sweat sensors [88] can also be utilized to detect the variations in related factors that are affected by hypoxic responses such as anxiety levels, respiratory rates and the fraction of exhaled nitric oxide and carbon monoxide.

To integrate these physiological parameters, flexible multifunctional sensors offer a unified measurement platform with compactness and lightweight benefits [89]. Specifically, stretchable laser-induced graphene-based sensors showed strength in simultaneously tracking various physiological signals (e.g. ECG, EOG, EMG, EEG, temperature and respiratory rate) [90,91], which not only enhances the accuracy and efficiency of data measurements, but also promotes user comfort and long-term wearability. Although the effectiveness of wearable sensors in physiological monitoring has been primarily evaluated based on various datasets from an algorithm development perspective [92,93], challenges still remain in addressing the inaccuracies that arise from intersubject variability and special groups (e.g. pregnant women and newborns).

### Pre-acclimatization

Pre-acclimatization is a process that helps the body to adjust to increased altitudes through physiological changes, characterized by enhancing ventilation to counteract reduced oxygen availability, usually performed in real or simulated environments. The related term ‘staging’ refers to spending a few days at moderate altitudes, allowing the body to undergo partial adaptation for further rapid exposures to higher elevations.

#### Pre-acclimatization strategies

A number of existing studies have analysed the effectiveness of their self-designed ‘staging’ strategies with different experimental settings, among which continuous residence at 2000–3000 m for 6–7 days was widely adopted for subsequent ascent to 4300 m [94]. A short stay for 1–2 days was also considered to be effective in reducing AMS risks [95]. Related factors such as target altitudes and physical activity affect the AMS prevalence as well [96] and have been proven to be crucial for subsequent ascent. Another concept called slow ascent (sometimes regarded as a type of staging) advises a gradual increase in altitude, particularly above 2500 m, with a recommended daily ascent rate that does not exceed 500 m [97]. This careful progression is essential for mitigating the risks of altitude-induced ailments and includes taking rest days after every 1000–1500 m of ascent, to ensure a more efficient and safer acclimatization process. Additional rules for pre-acclimatization can be found [98].

Rather than physically presenting at elevated altitudes, artificial exposure approaches allow individuals to intermittently stay under the simulated hypoxic conditions in controlled settings, which are incorporated into daily routines and offer practical and accessible options for pre-acclimatization. A commonly used strategy is intermittent hypoxic exposure (IHE), which exposes individuals to hypoxia conditions for multiple sessions with recovery intervals in normoxia or hyperoxia [99].

For different target altitudes, protocols with various hypoxic exposure intensities have been proven to be efficient [100–103]. The strategy of 4 h of pre-exposure for 7 or 15 d in simulated hypobaric environments was shown to be feasible in reducing AMS at 4300 m [101], supported by the observations of decreased LLS and increased SpO_2_. A recent study reported the effectiveness of 4 h of pre-exposure (12% FiO_2_) acclimatization to 3250 m, which curtailed hypoxia-induced inflammation and dyslipidemia [102]. Although short-period IHE (e.g. 1 h per day for a week) is also useful in increasing HVR and lowering AMS severity, the improvement may not be significant for very high altitudes (>3500 m) [104,105]. Therefore, long-term hypoxic exposure periods are required to achieve more persistent and beneficial effects. Besides, the efficacy differences of normobaric and hypobaric hypoxia were also analysed in a number of studies, most of which reported more significant physiological acclimatization (higher resting ventilation, larger desaturation and increased periodic breathing) under the hypobaric condition [106]. In spite of the potential of pre-acclimatization, some conflicting results exist in these findings. This may be attributed to different experimental settings and intersubject variability, which are the main challenges for determining the optimal pre-acclimatization strategies and call for the need for personalized pre-acclimatization.

In addition, remote ischemic preconditioning (RIPC) is also a promising pre-acclimatization approach for high altitudes. RIPC induces the local repetitive ischemia and reperfusion of limbs by inflating and deflating BP cuffs, in order to activate the protective physiology of the human body and then reduce the following ischemic and hypoxic injury of remote organs [107,108]. The restriction of blood flow and transportation caused by periodic ischemia can result in intermittent hypoxia, so RIPC may lead to similar physiological effects to IHE. Common benefits include accelerated cognitive functional recovery [108], enhanced oxygenation capacity [109] and increased HIF-1$\alpha $ levels [110]. The effectiveness of various paradigms of RIPC in reducing risks of high-altitude illnesses has been evaluated and discussed. Most studies selected an intervention protocol with 5 min of ischemia followed by 5 min of reperfusion and RIPC with different durations (4 weeks, 1 week, less than a week) has been proven to be useful in high-altitude protection. Nevertheless, the outcomes of IHE and RIPC depend on their intensity. Moderate IHE and RIPC are generally not associated with adverse effects, whereas the excessive hypoxia and blood flow restriction that are caused by IHE and RIPC, respectively, may pose significant risks. In addition, there is an ongoing debate on the role and protocol design of RIPC in preventing AMS. For instance, in [111], a short-term RIPC protocol that was composed of four cycles of lower-limb ischemia was performed within 30 min before ascent, but no significant effects were found in preventing AMS; a similar result was also reported by [112]. Similar bottleneck issues (to the case of IHE) remain in personalized optimal RIPC design; in particular, the way of determining optimal ischemia duration, frequency and magnitude varies across different scenarios and remains to be systematically explored.

#### Closed-loop control for pre-acclimatization

IHE is becoming increasingly popular for its ease of adjustment and higher safety, as well as RIPC. However, the huge heterogeneity in experimental settings prevents the exploration of optimal pre-acclimatization strategies. Current protocols are primarily based on expert experience, in which the hypoxia and ischemia dose, duration and frequency of each episode and the number of training days are the main factors to be designed. These factors comprehensively decide the simulated ascending rate and their influence on adaptation is validated in different ways. Bustcher *et al.* observed a 4.5-fold steeper increase in the incidence of AMS for air travel compared with slower ascent modes (hiking or combined car) based on 12 studies with 11 021 individuals who were ascending to 19 different altitudes [113].

As mentioned above, although a moderate strategy facilitates the adaptation to reduced oxygen availability, a severe hypoxia dose can result in pathological disorders [114]. Yu concluded that the AMS happens when the hypoxic environment changes too rapidly for the body's self-organizing processes to compensate for it and maintain homeostasis [115], which can be expressed as ‘environment variation rate (EVR) >> physical self-organizing and self-adapting rate (PSSR)’. In other words, the key to improving adaptation is to determine the proper induced hypoxia dose that guarantees EVR $\ge $ PSSR, but the definition of ‘proper’ is quite different for each individual due to the large inter- and intrasubject variability in high-altitude adaptability (reflected in the PSSR). Some individuals may fall sick at a slower ascendspeed whereas others can move faster than the speed that is recommended in the guidelines without experiencing any difficulty. Besides, the problem of tolerating higher altitudes was observed for people who quickly acclimated to moderate altitudes whereas those who needed more time for low-altitude adaptation found it easy to adapt to the subsequent extreme altitudes [116]. In this regard, it is hard to find a single optimal strategy that is suitable for all individuals.

From a systems and control perspective, the pre-acclimatization procedure is essentially a dynamic regulation process of the hypoxia-related physiological states, which can be achieved through real-time performance monitoring, system identification and feedback control to achieve a safe and suitable homeostasis level before entering the targeted ascend altitude. This observation plays an important role in the research of personalized pre-acclimatization. Specifically, by treating the physiological process that is affected by hypoxia as a dynamic system, the hypoxia exposure that is determined by the pre-acclimatization protocol as inputs and the measurable physiological parameters as outputs, the system output response depends not only on the inputs, but also on the internal structure and dynamic characteristics of the system. In this sense, the design of the pre-acclimatization strategy should consider not only the pre-acclimatization requirements (such as the target altitude), but also the characteristics and changes in the hypoxic responses of the human body. This motivates the idea of achieving personalized pre-acclimatization under the framework of feedback control or, more specifically, physiological closed-loop control systems. In particular, closed-loop personalized pre-acclimatization is made possible by the clinical evidence of using physiological indicators to predict AMS risks and the use of IHE or RIPC to accelerate hypoxia pre-acclimatization; the availability of wearable physiological sensing devices and programmable hypoxicators and RIPC devices; and, more importantly, the essential robustness and effectiveness of the state-of-the-art learning-based control algorithms [117–119] that do not rely on exact and potentially complex physiological models. Physiological parameter monitoring (e.g. SpO_2_ and HR) is a critical component for personalized closed-loop pre-acclimatization, as it not only participates in the characterization of hypoxic responses of individuals, but also provides feedback information for the automated regulation of hypoxia settings (low oxygen concentration, duration of low-oxygen intervals) or ischemia doses (cuff pressure, inflation/deflation cycles).

Considering the fact that IHE and RIPC involve several days or weeks of exposure to hypoxia or ischemia and that the adaptation process of the human body is relatively slow, run-to-run control, which is a systematic and effective approach that is utilized for slow and batch processes in chemical process control [120], can be tailored for the pre-acclimatization procedure considering its natural day-to-day cycle. In run-to-run control, the parameters for the next run are updated based on the results of the previous production run; for personalized pre-acclimatization, the manipulated variables such as oxygen concentration and training duration can be dynamically regulated based on a run-to-run control law, according to the performance of the previous training session. Compared with the current constant predesigned protocols during the entire pre-acclimatization process, personalized run-to-run solutions enable dynamic feedback-based adjustment of training parameters for enhanced safety and performance. With the abundance of data obtained from wearable devices, more advanced data-driven learning-based control approaches can be utilized. For instance, a Bayesian optimization method with safety constraints was proposed to enable the optimal decision of low oxygen concentration for pre-acclimatization [13], based on the comprehensive indicator that was constructed by using dynamic SpO_2_ data [14], although its effectiveness needs to be further validated based on clinical results. Note that existing literature on closed-loop drug delivery and feedback control approaches, e.g. proportional-integral-derivative control and model predictive control, has demonstrated their safety and effectiveness in large clinical studies [121,122].

To generate artificial hypoxic conditions for closed-loop pre-acclimatization, hypoxicators such as normobaric/hypobaric hypoxia chambers, tents or mask systems have been designed. In these systems, the low air pressure is achieved by reducing the atmospheric pressure within enclosed chambers and the low oxygen is obtained through gas-separation and gas-mixing approaches. For gas separation, pressure swing absorption (PSA) [123] and membrane separation [124] are widely used to extract nitrogen from ambient air, thereby reducing the oxygen concentration. The former extracts nitrogen by using molecular sieves and the latter uses polymetric material as a membrane to separate the oxygen and nitrogen according to the differences in permeability. Gas mixing, on the other hand, creates hypoxic conditions by blending ambient air with low-oxygen gases, which offers greater control over oxygen levels.

### Prospects

#### Real-time physiological parameter monitoring

How to accurately and automatically obtain the physiological state of an individual is the first question that we confront in developing personalized pre-acclimatization protocols. With the development of wearable sensors and the Internet of Medical Things, it has become possible to explore novel indexes to predict AMS risk or new indicators of hypoxic damage in a data-driven fashion by associating the gold standards in clinical diagnosis with the underlying features in the continuously monitored physiological data (e.g. SpO_2_, HR, sleep quality). The feasibility and potential of this approach have been investigated in designing key performance indicators for AMS risks with SpO_2_ data obtained during pre-acclimatization [14], but it is not yet known how to find more effective prediction indexes with multivariable physiological data for more challenging scenarios by using state-of-the-art ML and data-mining approaches. Finally, joint effort from clinicians and engineers is needed to conduct the appropriate cohort studies for data generation and to design intelligent soft-sensing algorithms.

#### Feedback regulation of physiological parameters with safety guarantee

Intelligent and autonomous control seems to be an ideal enabling technology for personalized health management during the pre-acclimatization phase, with the help of real-time physiological parameter monitoring. Advanced feedback-based decision algorithms can be used to design optimal pre-acclimatization protocols that are adjusted based on the training status of an individual. Learning-based control approaches can be adopted to dynamically and autonomously understand the change in the underlying physiological dynamics, which will help to ensure the safety and effectiveness of the intelligent decision algorithms. On the other hand, it is essential to involve clinicians in this process and provide constructive clinical suggestions. Clinicians are not only encouraged to directly participate in the clinical studies, but also indirectly assist in the design and improvement of the closed-loop control algorithms, which further ensures the safety and enhances the interpretability of closed-loop algorithms, and thus quickly promotes the subsequent clinical application.

#### Medical digital twin for *in silico* validation

**Figure 7.**
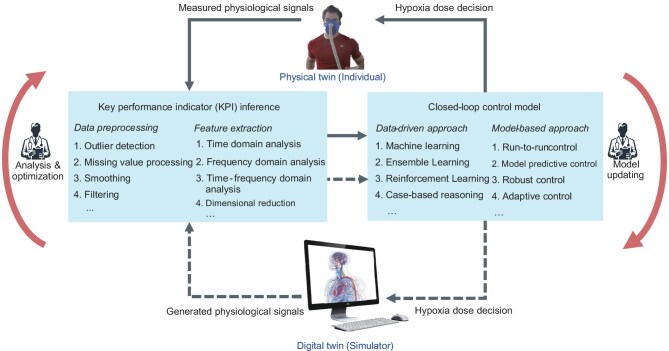
Flowchart of medical digital twin for pre-acclimatization algorithm development. The key performance indicator is generally obtained after data preprocessing and feature extraction processes, and it is used for subsequent decision-making. The closed-loop control model can be established by using run-to-run and artificial intelligence approaches. In the early stage of algorithm development, the recommended hypoxia dose given by the model is first transmitted to the simulator for *in silico* validation. After analysis and optimization, the improved algorithm is used in real clinical studies and the collected clinical data can be further utilized to update the digital physiological model.

Another obstacle to achieving personalized pre-acclimatization is the difficulty in conducting massive clinical trials in order to understand the clinical evidence and test the personalized treatment strategies, which consume a large amount of time, resources and manpower. Besides, trials in some harsh scenarios can also bring great physiological and psychological risks to the subjects. With the development of digital technology and the availability of physiological datasets, mathematical models that describe physiological responses to hypoxia can be used in computer algorithms to create *in silico* subjects and dynamically simulate their physiological changes under different hypoxia scenarios, which would be a helpful way to accelerate preliminary clinical validation and is also known as a medical digital twin. A flowchart of a medical digital twin for pre-acclimatization algorithm development is given in Fig. 7, which constructs a digital model of specific physiological processes through multimodal data of the human body; the physiological states under the IHE protocol can be simulated continuously to evaluate the performance of the protocol and achieve precision medicine [125] (namely, personalized IHE).

## HEALTH MANAGEMENT AT HIGH ALTITUDES

Although suitable pre-acclimatization can reduce the risk of severe AMS after entering high altitudes, the importance of high-altitude health management can never be overstated for long-term physical damage prevention, for both high-altitude residents and travelers who stay at high-altitude locations for long periods. In this section, we review the advances in long-term health monitoring and management at high altitudes.

### CMS diagnosis and physiological monitoring

CMS is a syndrome that is characterized by excessive erythrocytosis (EE) and typically occurs in high-altitude residents (>2500 m). The presence of EE is defined as a hemoglobin (Hb) concentration of >21 g/dL for males and >19 g/dL for females [126]. Symptoms that include headache, breathlessness, palpitations, tinnitus, paresthesia, dilatation of veins, cyanosis and sleep disturbances are also associated with CMS diagnosis. These signs are quantified through the Qinghai CMS score [126]. People with a score of >5 are diagnosed as having CMS (mild CMS: 6–10, moderate: 11–14, severe: >15). Besides, severe hypoxemia is also considered as a criterion in some scoring systems, but the cut-off values vary in different regions (Peru at 4340 m: <83% or <81.5% [127], Qinghai–Tibet plateau: <85%) [128].

For people who work at high altitudes but live at sea level (e.g. mine workers), a new condition named long-term chronic intermittent hypoxia may appear because of the work-shift system, which typically involves 4–7 d for work and rest in turn and puts them in an intermittent hypoxic environment, causing remodeling of the cardiac and pulmonary vasculature and a reduction in working capability [129]. Highlanders may also suffer from CMS, as pregnant highlanders and their newborns face more severe survival challenges. The abnormal uterine vasodilator responses that are caused by chronic hypoxia limit elevation in the uterine artery blood flow of pregnant highlanders, which is a contributing factor to the deceleration of fetal growth. Besides, the cerebral oxygen saturation (rScO_2_) of newborns at high altitudes is significantly lower than those at sea levels [130], which may result in subsequent brain injury if not found and prevented in time. Therefore, the implementation of real-time physiological monitoring has become imperative. Large-scale clinical trials on the use of wearable devices in pregnant women and newborn health management are ongoing [131–133], which can further advance the adoption of monitoring technologies.

However, environments that are characterized by hypoxia, low temperatures and low pressure present unique challenges for the design of wearable devices, as the physiological signals (e.g. PPG) become different or blurred compared with the corresponding signals at low altitudes. Several efforts have been devoted to wearable system design for physiological parameter monitoring at high altitudes. For instance, for high-altitude utilization (>2000 m), a wearable T-shirt that integrates several non-invasive sensors to measure HR, respiration rate, SpO_2_, body and ambient temperatures, and relative humidity levels that can be transmitted wirelessly to a central monitoring station has been developed [134]; an effective response to low-intensity physical activities for both a controlled laboratory scenario and a real work scenario was found [135]. NASA developed a body-worn system called Lifeguard based on the analysis of 24 application scenarios to achieve multiparameter monitoring in extreme environments, which could remotely transmit the vital signals from Licancabur volcano in Chile (∼6000 m) to Stanford, NASA in real time [136].

### Automated oxygen delivery

Once the abnormal physiological state is detected, the most direct and effective way to relieve discomfort for individuals who cannot immediately descend to sea level is for them to inhale oxygen [6]. To ensure effective oxygen therapy, different oxygen-supply criteria have been proposed, which provide guidelines for administering oxygen in various settings. For the construction personnel of high-altitude tunnels, criteria that focus on labor capacity and life support, respectively, were proposed in [137], which was the first study to have considered the joint impact of labor intensity and oxygen consumption at different altitudes; at altitudes of 3000–5000 m, individuals with abnormal Hb levels or significant symptoms (normal Hb concentration but a Qinghai CMS score of >5) received daily oxygen supplementation, with each session lasting 1 h.

The traditional way to supply oxygen is through the nasal inhalation method by using a high-pressure oxygen cylinder, which is suitable for outdoor work and activities. However, users have to replace the cylinder frequently due to its limited storage capacity. In comparison, oxygen concentrators directly extract oxygen from ambient air, which enables continuous oxygen delivery for long-term use at home or in clinical settings [138]. For severely hypoxic patients who need large amounts of oxygen, a liquid oxygen (LOX) device is often a better choice. It not only takes up less space than oxygen in its gas form, but also provides a higher oxygen concentration. Accessory tools such as masks and tents are connected to these devices for oxygen delivery.

Apart from devices that are used for individual oxygen therapy, some specialized systems have been established in high-altitude settings. For instance, the Atacama large millimeter array—an astronomical interferometer that was built in northern Chile at 5050 m—generates oxygen through PSA and LOX. Another example is the construction of the Qinghai–Tibet railway in China, along which diffused and centralized oxygen-supply systems were equipped. The diffused oxygen-supply system controls the oxygen concentration of each carriage to between 23.5% and 25%, and the optimal concentrations can be automatically regulated as the altitude increases. In order to meet personalized requirements, the passengers can inhale the oxygen with higher concentration provided by the centralized oxygen-supply system, through independent interfaces or masks. In addition, oxygen-supply systems need to be widely arranged in large facilities, such as hospitals, schools and construction sites, which will significantly benefit the visitors and sojourners who live or work for different time durations.

Advanced oxygen generation technologies can be categorized into physical approaches and chemical approaches. Physical approaches include PSA, membrane separation and cryogenic air separation. Unlike hypoxicators that create low-oxygen conditions, PSA and membrane separation approaches are used in portable oxygen concentrators to enrich oxygen from the surrounding air. For cryogenic air separation [139], oxygen is extracted by liquefying air components under low temperatures, which is more effective in large-scale applications. Considering the advantage of low energy consumption, chemical approaches [140] have also been widely explored in high-altitude oxygen generation. The oxygen is released through the electrolysis of water or other chemical reactions that break down oxygen-containing compounds. All these technologies provide versatile solutions to meet oxygen-supply demands in remote high-altitude environments, which has accelerated the development of various oxygen-supply systems that are utilized in different scenarios.

### Prospects

#### Closed-loop regulation and dynamic scheduling for personalized oxygen supply

In certain high-altitude application scenarios, the limited availability of oxygen and power resources creates substantial difficulties in maintaining continuous and abundant oxygen supply for individuals. To maximize the duration of life support, closed-loop oxygen regulation can serve as a feasible solution. This entails the personalized modeling of physiological processes to characterize the dynamic hypoxic responses, which enables direct or indirect estimation of the oxygen demand. Achievement of this goal necessitates the application of advanced data-driven learning techniques, which facilitate the subsequent design of closed-loop control strategies under multiple resource constraints. Moreover, oxygen scheduling among individuals is a very relevant but more challenging problem. Mathematically, it is essentially a multi-objective adaptive optimization problem under complex constraints, with the consideration of intersubject physiological differences, limited resource availability and varying activity demands. ML techniques—particularly reinforcement learning—can offer a viable solution by enabling the continuous adaptation of oxygen scheduling strategies that are based on real-time feedback from the environment and individual physiological states. These scheduling strategies strike a better balance between competing objectives, to ensure effective and reliable decision-making for oxygen supply.

#### Cloud-based platforms for high-altitude healthcare

Enabled by the Internet of Medical Things and wireless communication, cloud-based platforms can provide comprehensive services for personalized physiological monitoring and intelligent intervention. A cloud-based healthcare platform integrates heterogeneous physiological data from multiple sources, based on which advanced artificial intelligent algorithms are utilized to extract key features for real-time remote health management. Besides, early alarm and telemedicine support systems can also be integrated to ensure the safety of the closed-loop intervention process. However, challenges also exist in the implementation of cloud-based healthcare platforms, particularly from the aspect of limited communication resources in rural areas. To confront the network instability in remote high-altitude regions, edge computing [141] and event-triggered data transmission [142] are promising options, which can minimize the reliance on consistent communications through localized data processing and intermittent data transmission, respectively. In addition, the security and privacy of the physiological data transmission should also be ensured, which can be achieved through leveraging the advanced encryption standard, which is a widely used encryption technique to secure information [143].

## CONCLUSION

Despite the encouraging and fruitful advances in pre-acclimatization and long-term high-altitude management, further enhancement of effective healthcare support and management measures to guarantee the safety of ever more frequent human activities at high altitudes is still urgently needed. Although the effectiveness of pre-acclimatization was demonstrated a few decades ago, it is still at a relatively early stage, with a lack of clear guidelines for hypoxia conditioning and conclusive results to demonstrate hypoxia conditioning robustness [7]. For high-altitude health support, continuous oxygen support seems to be the only available remedy to maintain a healthy physiological state at high altitude, which is not always available, depending on the type of activities in which people are involved.

Thanks to the rapid expansion of our knowledge in life sciences, medicine and engineering, high-altitude health management has gradually evolved into an interdisciplinary area. Through the joint effort of clinicians, scientists and engineers, personalized healthcare at high altitudes will ultimately step into real life, with cutting-edge technology in autonomous intelligent control as an enabling catalyst.
